# Novel insights into mutual regulation between N^6^-methyladenosine modification and LncRNAs in tumors

**DOI:** 10.1186/s12935-023-02955-1

**Published:** 2023-06-26

**Authors:** Nan Zhang, Yifei Sun, Zongqin Mei, Zuoshun He, Shiyan Gu

**Affiliations:** School of Public Health, Institute of Preventive Medicine, Dali University, No. 22, Wanhua Road, Dali, 671000 Yunnan People’s Republic of China

**Keywords:** N^6^-methyladenosine, LncRNAs, Methyltransferases, Demethylases, Methyl-binding proteins

## Abstract

N^6^-methyladenosine (m^6^A), one of the most common RNA methylation modifications, has emerged in recent years as a new layer of the regulatory mechanism controlling gene expression in eukaryotes. As a reversible epigenetic modification, m^6^A not only occurs on mRNAs but also on Long non-coding RNAs (LncRNAs). As we all known, despite LncRNAs cannot encode proteins, they affect the expression of proteins by interacting with mRNAs or miRNAs, thus playing important roles in the occurrence and development of a variety of tumors. Up to now, it has been widely accepted that m^6^A modification on LncRNAs affects the fate of the corresponding LncRNAs. Interestingly, levels and functions of m^6^A modifications are also mediated by LncRNAs through affecting the m^6^A methyltransferases (METTL3, METTL14, WTAP, METTL16, etc.), demethylases (FTO, ALKBH5) and methyl-binding proteins (YTHDFs, YTHDCs, IGF2BPs, HNRNPs, etc.), which are collectively referred to as “m^6^A regulators”. In this review, we summarized the mutual regulation mechanisms between N^6^-methyladenosine modification and LncRNAs in cancer progression, metastasis, invasion and drug resistance. In detail, we focus on the specific mechanisms of m^6^A modification, which is mediated by methyltransferases and demethylases, involves in the regulation of LncRNA levels and functions in the first part. And section two intensively displays the mediation roles of LncRNAs in m^6^A modification via changing the regulatory proteins. At last part, we described the interaction effects between LncRNAs and methyl-binding proteins of m^6^A modification during various tumor occurrence and development.

## Background

Recently, the regulatory roles of epigenetic modifications in the occurrence and development of various diseases have attracted much attention [1]. To date, more than 170 RNA modifications have been identified and methylation modifications, including N^6^-methyladenosine (m^6^A), N^1^-methyladenosine (m^1^A), 5-methylcytosine (m^5^C), N^7^-methylguanosine (m^7^G), are the most concerned [2]. Among these methylation modifications, m^6^A is the most abundant and important RNA modification in mammals [3]. First discovered on mRNA in 1974, m^6^A modification refers to methylation of the adenosine base at nitrogen-6 position [4]. Nowadays, a large number of studies confirmed that m^6^A exists not only on mRNAs but also on different non-coding RNAs, including Long non-coding RNAs (LncRNAs) [5, 6]. It has been widely accepted that m^6^A modification is a reversible methylation modification and is dynamically catalyzed by methyltransferases, demethylases and methyl-binding proteins, which are collectively referred to as m^6^A regulators [7]. Specifically, methyltransferases, which mainly include methyltransferase-like 3 (METTL3), methyltransferase-like 14 (METTL14), Wilms’ tumor-associated protein (WTAP), methyltransferase-like 16 (METTL16) and so forth, catalyze the formation of m^6^A modification, while the demethylases including obesity-associated protein (FTO) and alkB homologue 5 (ALKBH5) erase the m^6^A modification. Methyl-binding proteins, which combine with m^6^A modification and mediate its biological function, generally included YT521-B homology (YTH) domain family, insulin-like growth factor 2 mRNA-binding proteins (IGF2BPs) and heterogeneous nuclear ribonucleoproteins (hnRNPs) [4]. Under the simultaneous mediation of these regulatory proteins, RNA m^6^A modification widely involves in RNA generation, splicing, stabilization and degradation, thus participating in a variety of pathological and physiological functions [8]. Accumulating studies indicated that biological effects of m^6^A modification are closely associated with a variety of human diseases, particularly cancers, in which m^6^A modification can drive the progression, metastasis, invasion, and drug resistance of tumor cells [7, 9].

Long non-coding RNAs (LncRNAs) are transcripts of more than 200 nucleotides which generally lack protein-coding potential, while they could perform distinct biological functions through interacting with RNAs or proteins [10]. There are currently 172,216 human and 131,697 mouse transcripts annotated in the systematic NONCODEv5 database and 270,044 human LncRNAs in the curated knowledgebase LncBook, making LncRNAs become the most diverse class of regulatory ncRNAs [11]. At present, LncRNAs are abnormally expressed in various tumors and are widely accepted as desirable biomarkers for indicating progression, metastasis and invasion processes of cancer cells [12]. Recently, accumulating studies have found that LncRNAs were modified by m^6^A modifications, which conversely affect processing and function of corresponding LncRNAs [13]. In detail, m^6^A modification not only affects the cleavage, stability and degradation of corresponding LncRNAs but also influences the interactions between LncRNAs and miRNAs, mRNAs or proteins [14–16]. Interestingly, emerging researches have demonstrated that LncRNAs have critical roles in regulating m^6^A modification through mediating its methyltransferases demethylases and methyl-binding proteins [17–19]. At present, the mutual regulation between m^6^A modification and LncRNAs has attracted a lot of attention in tumor occurrence and development. Thus, in this review, we thoroughly summarized the recent advances of m^6^A modification on LncRNAs in the modulation of cancer cell progression, metastasis and invasion, as well as the mediation roles of LncRNAs in m^6^A modification levels and functions through affecting m^6^A regulators during the occurrence and development of various tumors, indicating that targeting the interaction between m^6^A modification and LncRNAs is expected to become a desirable strategy for cancer prevention and treatment.

## LncRNAs regulated by m^6^A modification in various tumors

As mentioned above, m^6^A modification on LncRNAs has been well established to mediate the functions of corresponding LncRNAs. Recently, accumulating studies have confirmed that m^6^A modification, which is mediated by METTL3, METTL14, WTAP, FTO and ALKBH5, the widely known methyltransferases and demethylases, on LncRNAs can affect the level and biological function of corresponding LncRNAs, thus involving in the occurrence and development of various tumors, which originate from respiratory system, digestive system, reproductive system and so forth [9–61].

### m^6^A-modified LncRNAs mediated by methyltransferases

#### METTL3 mediates the m^6^A modification on LncRNAs

As a dynamic and reversible modification, m^6^A methylation is catalyzed by methyltransferase complex, in which METTL3 serves as the main catalytic core [9]. At present, METTL3 has been well evidenced to mediate various LncRNAs through catalyzing m^6^A modification. For example, the stability of LncRNA FAM225A is strengthened by m^6^A modification, which mediated by METTL3, thus up-regulating LncRNA FAM225A level in nasopharyngeal carcinoma cells. Mechanistically, FAM225A functioned as a competing endogenous RNA (ceRNA) for sponging miR-590-3p and miR-1275, leading to the upregulation of their target integrin β3 (ITGB3), finally activation of focal adhesion kinase (FAK)/phosphoinositide 3-kinase (PI3K)/protein kinase B (AKT/PKB) signaling to promote nasopharyngeal carcinoma cells proliferation and invasion [16]. In addition, LncRNA small nucleolar host gene 1 (SNHG1) transcript was also modified by METTL3-mediated m^6^A modification, thus improving the stability of LncRNA SNHG1 and decreasing the rate of RNA degradation, which leads to upregulation of LncRNA SNHG1 in non-small cell lung cancer (NSCLC). Further, LncRNA SNHG1, as a competing endogenous RNA, was able to sponge miR-140-3p to increase ubiquitin-conjugating enzyme endometrial cancer consortium (E2C2) expression in NSCLC cell lines [5]. Interestingly, small nucleolar host gene 17 (SNHG17), a LncRNA homologous to SNHG1, has been also shown to play a regulatory role and closely relate to m^6^A modification in lung adenocarcinoma progression. Mechanistically, METTL3-mediated m^6^A modification could induce the upregulation of LncRNA SNHG17 by enhancing its RNA transcript stability [20]. Also in lung adenocarcinoma cells, the highly expressed LncRNA AC098934 facilitates the cell proliferation as well as invasion either in vitro or in vivo. Further exploration data displayed that LncRNA AC098934 promoted the malignant behavior of lung adenocarcinoma cells under the m^6^A modification induced by METTL3 [21]. Metastasis-associated lung adenocarcinoma transcript 1 (MALAT1), a LncRNA highly correlate to the occurrence and development of lung cancer, has been also indicated that its level was increased due to a higher level of m^6^A modification mediated by METTL3 and meanwhile, METTL3/YTHDF3 complex is able to elevate the stability of MALAT1 in non-small cell lung carcinoma (NSCLC) cells [22]. Similar to that in lung adenocarcinoma, LncRNA MALAT1 is also methylated and delocalized by METTL3 in Thymic epithelial tumors [23]. Besides, LncRNA Lung Cancer Associated Transcript 3 (LCAT3) was also found to be up regulated in lung adenocarcinomas and its over-expression was closely associated with the poor prognosis of lung adenocarcinoma patients. Regarding the regulatory mechanisms, METTL3-mediated m^6^A modification on LncRNA LCAT3 transcript can enhance its stabilization and upregulate its level [24]. Similarly, the enhanced stability of LncRNA ABHD11 antisense RNA 1 (ABHD11-AS1), which ectopic overexpression closely associated with unfavorable prognosis of non-small cell lung carcinoma patients, was also manipulated by METTL3-mediated m^6^A modification and thus upregulation of LncRNA ABHD11-AS1 level [25]. In addition, high expression of LncRNA SVIL-AS1, of which expression is also upregulated by METTL3-mediated m^6^A modification, can promote the occurrence and development of lung adenocarcinoma [26]. Another study indicated that the level of LINC00958, which independently predicted poor overall survival of hepatocellular carcinoma patients, was also elevated by METTL3-mediated m^6^A modification [27]. In addition, LncRNA THAP7-AS1 has been detected as showing high expression and correlating with positive lymph node metastasis and poorer prognosis of gastric cancer. Further exploring results revealed that METTL3 catalyzes the formation of m^6^A modification on LncRNA THAP7-AS1 and enhanced its expression depending on the "reader" protein IGF2BP1 [28]. In colorectal cancer cells, Wu et al. found that LncRNA RP11 level was enhanced by METTL3-mediated m^6^A methylation and positively regulated the migration, invasion and epithelial mesenchymal transition (EMT) of colorectal cancer cells. Mechanistically, heterogeneous nuclear ribonucleoprotein A2/B1 (hnRNPA2B1), as well known m^6^A methyl-binding protein, formed a complex with LncRNA RP11 to mediate the degradation E*3 ligases*, *Siah1* and *Fbxo45* mRNA in an m^6^A-dependent manner, thus preventing the proteasomal degradation of Zinc finger E-box-binding homeobox (ZEB1), of which upregulation was essential for LncRNA RP11-induced cell dissemination [15]. In addition, high expression of LncRNA plasmacytoma variant translocation 1 (PVT1) made prostate cancer cells more proliferative, migratory and invasive, whereas LncRNA PVT1 knockdown led to the opposite phenotype. In terms of specific mechanisms, the expression of LncRNA PVT1 upregulated by METTL3-mediated m^6^A modification on LncRNA PVT1, which subsequently sequestered miR-27b-3p within cells, thereby indirectly promoting the bloom syndrome protein expression [29].

#### METTL14 mediates the m^6^A modification on LncRNAs

METTL14, another important catalytic enzyme for m^6^A modification, has also been well established to regulate m^6^A modification on different LncRNAs in various cancers, including head and neck squamous cell carcinoma, oral squamous cell carcinoma, lung adenocarcinoma, colorectal cancer, renal cell carcinoma, hepatocellular carcinoma and so forth [30–38]. In detail, LncRNA LNCAROD overexpressed in head and neck squamous cell carcinoma and shortened overall survival of patients. The dysregulation of m^6^A modification on LncRNA-activating regulator of DKK1 (LNCAROD), which was mediated by METTL3 and METTL14, might account for enhancing the LNCAROD stability and elevating its level, thus promoting proliferation, mobility and tumorigenicity in head and neck squamous cells [30]. Li et al. indicated that both METTL14 and LncRNA metastasis-associated lung adenocarcinoma transcript 1 (MALAT1) were elevated in oral squamous cell carcinoma tissues and cells. Silencing METTL14 repressed OSCC cell viability and colony formation while excitation of LncRNA MALAT1 was able to reverse the inhibition effects of silencing METTL14. Further experimental data demonstrated that METTL14 induced m^6^A modification on LncRNA MALAT1 thus increasing the level of LncRNA MALAT1, which acts as a sponge to absorb miR-224–5 and promote histone lysine demethylase 2A transcription [31]. In addition, LncRNA human leukocyte antigen complex group 11 (HCG11) served as a tumor suppressor to restrain tumor growth in lung adenocarcinoma. Mechanistically, the METTL14-mediated m^6^A modification on LncRNA HCG11 enhanced its nuclear exportation, thus recruiting IGF2BP2 to target *Large Tumor Suppressor Kinase 1* (*LATS1*) mRNA to enhance the stability and promote the translation of *LATS1*, finally influencing the growth of lung adenocarcinoma [32]. The study from Yang et al. indicated that knockdown of METTL14 drastically enhanced proliferative and invasive ability in colorectal cancer cells. Further exploring data identified that LncRNA X inactive-specific transcript (XIST) was the downstream target of METTL14, of which low expression significantly attenuated m^6^A modification on LncRNA XIST and augmented LncRNA XIST expression through attenuating degradation [33]. In addition, studies have revealed that LncRNA ZFAS1/RAB22A in atherosclerosis [34], LINC01320 in gastric cancer [35], Lnc-LSG1 and LncRNA NEAT1 in renal cell carcinoma [36, 37], LncRNA MIR155HG in hepatocellular carcinoma were also regulated by METTL14-mediated m^6^A modification [38], indicating that METTL14 can regulate the modification of m^6^A on various LncRNAs, therefore participating in the genesis and development of various tumors.

#### WTAP mediates the m^6^A modification on LncRNAs

As a kind of adaptor protein of m^6^A methyltransferase complex, WTAP has been well established to involving in the mediation of m^6^A modification on LncRNAs, such as LncRNA DIAPH1-AS1 [39], LncRNA FOXD2-AS1 [40]. In detail, WTAP-mediated m^6^A level on DIAPH1-AS1 can enhance the stability of DLGAP1-AS1 in an IGF2BP2-dependent pathway, thus participating in the growth and metastasis of naso-pharyngeal carcinoma cells [39]. In addition, m^6^A-modified LncRNA FOXD2 adjacent opposite strand RNA 1 (FOXD2-AS1) has been also evidenced to involving in the occurrence and progression of osteosarcoma. Mechanistically, a remarkable number of m^6^A-modified sites were found on the 3'-UTR of FOXD2-AS1, thus enhancing the stability of FOXD2-AS1 transcript, on which m^6^A modification was promoted by WTAP [40].

#### Other methyltransferases mediate the m^6^A modification on LncRNAs

In addition to METTL3, METTL14 and WTAP, some RNA m^6^A modification methyltransferases, such as Zinc finger CCHC domain-containing protein 4 (ZCCHC4), KIAA1429 and METTL16, also catalyze m^6^A modification on LncRNAs [41–43]. In detail, ZCCHC4, one of latest identified m^6^A methyltransferases which primarily methylates human 28S rRNA and also interacts with a subset of mRNAs, was found to be inhibited DNA damage-induced apoptosis in hepatocellular carcinoma cells by interacting with LncRNA AL133467.2 Further exploring data revealed that knockout of ZCCHC4 promotes AL133467.2 and γH2AX interaction for enhancing chemosensitivity in hepatocellular carcinoma cells [41]. KIAA1429, another methyltransferase, was also involved in mediation of m^6^A modification on LncRNA LINC00958 in gastric cancer cells. Mechanistically, m^6^A-modified sites in LINC00958 have been identified by using methylated RNA immunoprecipitation sequencing (MeRIP-Seq). Moreover, KIAA1429 catalyzed the m^6^A modification on LINC00958, which interacted with *glucose transporter type 1* (*GLUT1)* mRNA in an m^6^A-dependent manner to strengthen the stability of *GLUT1* mRNA, thus promoting the gastric cancer cells' aerobic glycolysis [42]. In addition, originally thought to be a ribosomal RNA methyltransferase, METTL16 has now been shown to bind and methylate LncRNA RAB11B-AS1. In detail, METTL16 directly bound LncRNA RAB11B-AS1 and induced its m^6^A modification, which decreased the stability of LncRNA RAB11B-AS1 transcript thus resulting in the down-regulation of RAB11B-AS1. This reduction in LncRNA RAB11B-AS1 level caused by the elevation of METTL16 was correlated with poor prognosis of patients with hepatocellular carcinoma [43].

### m^6^A-modified LncRNAs mediated by demethylases

#### FTO mediates the m^6^A modification on LncRNAs

Recently, emerging studies have demonstrated that FTO was sincerely involved in the mediation of m^6^A modification on LncRNAs, thus participating in the occurrence and development of various tumors [44–46]. Results from Cui et al. indicated that the elevation of FTO expression demethylated m^6^A modification on LINC00022 transcript, thus hindering the LINC00022 degradation mediated by YTHDC2 in esophageal squamous cell carcinoma [44]. Han et al. systematically assessed the m^6^A modification expression of 407 gastric cancer clinical samples based on 23 m^6^A regulators and comprehensively associated these genes with LncRNAs. Importantly, LncRNA AC026691.1 could inhibit both migration and proliferation of gastric cancer through FTO demethylation [45]. In cervical cancer, LncRNA HOXC13 antisense RNA (HOXC13-AS) was increased and promoted the malignant phenotype of cervical cancer cells. Mechanistically, LncRNA HOCX13-AS1 expression was augmented Frizzled class receptor 6 (FZD6) by cAMP-response element binding protein-binding protein (CBP)-modulated histone H3 on lysine 27 acetylation (H3K27ac). Additionally, FTO reduced m^6^A and stabilized HOXC13-AS thus up-regulating FZD6 and activating Wnt/β-catenin signaling to drive cervical cancer cell proliferation, invasion and epithelial mesenchymal transition, suggesting HOXC13-AS as a potential target for cervical cancer treatment [46].

#### ALKBH5 mediates the m^6^A modification on LncRNAs

As a demethylase of m^6^A modification, ALKBH5 directly revers**es** m^6^A modification on adenosine [9]. Recently, some studies have revealed that ALKBH5 detaches m^6^A modification on various LncRNAs and involves in the occurrence and development of various tumors [47–60]. As we all known, LncRNA nuclear enriched abundant transcript 1 (NEAT1) is a carcinogenic LncRNA and its level and function were closely related to ALKBH5-demethylated m^6^A modification [47–51]*.* Literature from Dong et al. indicated that m^6^A-modified LncRNA NEAT1 was involved in glioblastoma multiforme. Specifically, hypoxia-induced ALKBH5 removed m^6^A modification from the LncRNA NEAT1, enhancing its transcript stabilization and promoting NEAT1-mediated paraspeckle assembly, ultimately generating an immunosuppressive tumor microenvironment [47]. According to predictions in bioinformatics, Zhang et al. indicated that LncRNA NEAT1 is a potential binding LncRNA of ALKBH5. Further detection results demonstrated that both LncRNA NEAT1 and ALKBH5 were overexpressed in gastric cancer cells and tissues. ALKBH5 influences the expression of NEAT1 through removing m^6^A modifications on LncRNA NEAT1, of which high expression can subsequently lead to the enhancer of enhancer of zeste homolog 2 (EZH2) elevation. Specifically, NEAT1 can function as a scaffold by interacting with EZH2 to regulate the expression of EZH2 downstream genes, of which dysregulation is associated with invasion and metastasis of gastric cancer cells [48]. As same in gastric cancer, ALKBH5 also could up-regulate LncRNA NEAT1 expression by inhibiting m^6^A enrichment on LncRNA NEAT1 in hepatocellular carcinoma [49] and in colon cancer [50]. In addition to digestive system tumors, the elevation of LncRNA NEAT1 expression promoted by ALKBH5 demethylation is also observed in infantile hemangioma [51]. ALKBH5-mediated m^6^A modification on LncRNAs also plays a key role in the development of respiratory system tumors. Results from Li et al. indicated that LncRNA potassium voltage-gated channel subfamily Q member 1 opposite strand 1 (KCNQ1OT1) could directly bind to Homeobox A9 (HOXA9) to further regulate the proliferation, invasion and metastasis of Laryngeal squamous cell carcinoma cells. Further exploring data showed that ALKBH5 mediates LncRNA KCNQ1OT1 expression in an m^6^A-YTHDF2 dependent manner [52]. In esophageal squamous cell carcinoma tissues, the expression of LncRNA cancer susceptibility candidate 8 (CASC8) was higher than that in the control tissues and positively associated with the poor prognosis of patients. Further mechanism exploring data showed that the stability of LncRNA CASC8 transcript was enhanced by ALKBH5 mediated m^6^A demethylation [53]. ALKBH5-demethylated m^6^A modification on LncRNA RMRP was also identified in lung adenocarcinoma tissues [54]. Further, the cell proliferation, metastasis and cell cycle progression regulated by LncRNA TP53TG1 were closely related to m^6^A modification in gastric cancer cells. In detail, there were multiple m^6^A modification sites on LncRNA TP53 target 1 (TP53TG1), of which stabilization and expression were reduced by ALKBH5-demethylated m^6^A modification [55]. As a special LncRNA, long intergenic non-coding RNA LINC02551, a bona fide m^6^A target of ALKBH5, was downregulated by ALKBH5 in an m^6^A dependent manner in hepatocellular carcinoma [56]. LncRNA KCNK15-AS1 was able to effectively arrest proliferation, migration and EMT in pancreatic cancer cells. Data from mechanical experiments revealed that ALKBH5 was verified to increase m^6^A demethylation of LncRNA KCNK15-AS1 to control its elevation [57]. Also in pancreatic cancer, LncRNA DDIT4-AS1 was identified as one of the downstream targets of ALKBH5 through m^6^A-RNA immunoprecipitation and RNA sequencing in combination with bioinformatics analysis. Further experimental data revealed that stabilization of LncRNA DDIT4-AS1 was maintained by m^6^A-modified sites, which is essential for recruitment of Hu-Antigen R (HuR) to LncRNA DDIT4-AS1 [58]. Investigation from Chen et al. revealed that ALKBH5 could associate with LncRNA PVT1 and suppress LncRNA PVT1 degradation via erasing m^6^A modification in osteosarcoma. Mechanically, ALKBH5 decreased the m^6^A modification level on LncRNA PVT1, thus repressing the interaction between YTHDF2 and LncRNA PVT1 [59]. As same in osteosarcoma, knockdown of ALKBH5 contributed to reducing the stability of LncRNA PVT1 in lung cancer cells [60].

**Fig. 1:**
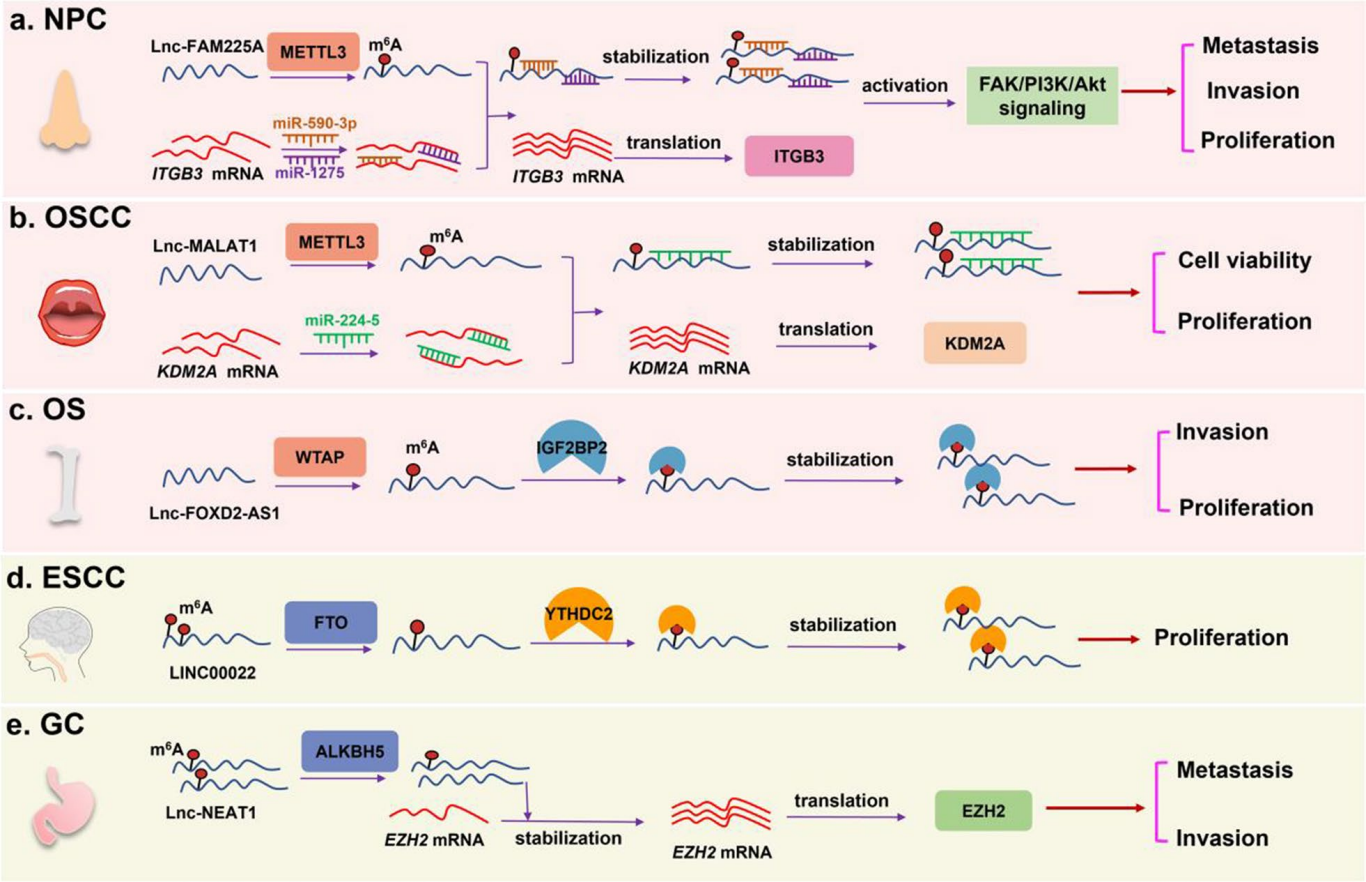
M^6^A modification and its methyltransferases and demethylases affected LncRNA function and level in various carcinogenic process. **a** In nasopharyngeal carcinoma (NPC), METTL3 up-regulated LncRNA FAM225A level thus promoting cells proliferation, invasion and metastasis. **b** In oral squamous cell carcinoma (OSCC), METTL14 increased LncRNA MALAT1 level, leading to elevating cell viability and proliferation. **c** In osteosarcoma (OS), WTAP enhanced LncRNA DIAPH1-AS1 stability, thus involving in the tumor cell growth and metastasis. **d** In esophageal squamous (ESCC), FTO inhibited LINC00022 decay, thus resulting in promoting cell proliferation. **e** In gastric cancer(GC), ALKBH5 reduced m^6^A level of LncR NEAT1, thereby promoting invasion and metastasis

In summary, accumulating studies have indicated that methyltransferases and demethylases influence the LncRNA levels and functions in various carcinogenic processes. We drew a schematic diagram, in which take METTL3 [16], METTL14 [31], WTAP [40], FTO [44] and ALKBH5 [48] as examples, to show the specific mechanisms of m^6^A modification involving in affecting LncRNA functions and levels (Fig. 1). Besides, the effects of m^6^A and its methyltransferases and demethylases on LncRNAs in different cancers are summarized and shown in Table 1.

**Table 1:** Regulation of m^6^A modification on LncRNAs in various tumors

Regulatory proteins of m^6^A	LncRNAs	Mechanisms of m^6^A mediating LncRNAs	Functions	Tumors	Refs.
METTL3**↑**	FAM225A**↑**	METTL3 up-regulated the level of FAM225A by enhancing the stability	Increased tumorigenesis and metastasis	Nasopharyngeal carcinoma	[16]
METTL3**↑**	SNHG1**↑**	METTL3 improved the stability of SNHG1 and decreased the rate of RNA degradation	Promoted proliferation	Non-small cell lung cancer	[5]
METTL3**↑**	SNHG17**↑**	METTL3 upregulated the SNHG17 level	Promoted proliferation, invasion, but inhibited apoptosis	Lung adenocarcinoma	[20]
METTL3**↑**	AC098934**↑**	METTL3 enhanced the AC098934 expression	Accelerated proliferation and malignant	Lung adenocarcinoma	[21]
METTL3**↑** YTHDF3**↑**	MALAT1**↑**	m^6^A modification enhanced the level and stability of MALAT1	Promoted metastasis	Non-small cell lung cancer	[22]
METTL3**↑**	MALAT1**↑**	METTL3 methylated and delocalized the MALAT1	Stimulated metastasis	Thymic epithelial tumours	[23]
METTL3**↑**	LCAT3**↑**	METTL3 enhanced the stability of LCAT3’s transcript	Promoted proliferation, migration, invasion, metastasis	Lung adenocarcinomas	[24]
METTL3**↑**	ABHD11-AS1**↑**	METTL3 upregulated the ABHD11-AS1 level by enhancing its stability	Promoted proliferation and Warburg effect	Non-small cell lung cancer	[25]
METTL3**↑**	SVIL-AS1**↑**	METTL3 upregulated the expression of SVIL-AS1	Stimulated proliferation	Lung adenocarcinoma	[26]
METTL3**↑**	LINC00958**↑**	METTL3 upregulated the level of LINC00958	Promoted proliferation	Hepatocellular carcinoma	[27]
METTL3**↑**	THAP7-AS1**↑**	-	Stimulated metastasis	Lymph node carcinoma	[28]
METTL3**↑** ALKBH5**↓**	RP11**↑**	m^6^A modification enhanced the stability of nascent LncRNA RP11 and elevated its expression	Stimulated migration, invasion and EMT	Colorectal cancer	[15]
METTL3**↑** METTL14**↑**	LNCAROD**↑**	m^6^A modification enhanced the stability of LNCAROD and elevated its level	Facilitated proliferation	Head and neck squamous cell carcinoma	[30]
METTL14**↑**	MALAT1**↑**	METTL14 increased the MALAT1 level	Increased cell viability and colony	Oral squamous cell carcinoma	[31]
METTL14**↓**	HCG11**↓**	METTL14 effected the stability and expression of LATS1	Promoted tumour growth	Lung adenocarcinoma	[32]
METTL14**↓**	XIST**↑**	METTL14 augmented XIST expression through reducing degradation	Promoted proliferative and invasive	Colorectal cancer	[33]
WTAP**↑**	DIAPH1-AS1**↑**	WTAP mediated m^6^A level on DIAPH1-AS1 and enhanced its stability	Stimulated metastasis	Nasopharyngeal carcinoma	[39]
WTAP**↑**	FOXD2-AS1**↑**	WTAP enhanced the stability of FOXD2-AS1 transcripts	Increased migration, proliferation	Osteosarcoma	[40]
ZCCHC4**↑**	AL133467.2**↑**	ZCCHC4 interacted with AL133467.2 to inhibit apoptosis	Repressed apoptosis	Hepatocellular carcinoma	[41]
KIAA1429**↑**	LINC00958**↑**	KIAA1429 catalyzing m^6^A-modified sites of LncRNA LINC00958	Increased proliferation	Gastric cancer	[42]
METTL16**↑**	RAB11B-AS1**↓**	METTL16 effected the level of RAB11B-AS1 by decreasing the stability	Facilitated proliferation, migration, and invasion, but repressed apoptosis	Hepatocellular carcinoma	[43]
FTO**↑**	LINC00022**↑**	FTO inhibited the decay of LINC00022	Facilitated proliferation and tumor growth	Esophageal squamous	[44]
FTO**↑**	AC026691.1**↑**	FTO interactied with LncRNA AC026691.1 and regulated its level	Accelerated migration and proliferation	Gastric cancer	[45]
FTO**↓**	HOXC13-AS**↑**	FTO-reduced m^6^A modification and contributed for stabilization HOXC13-AS	Facilitated proliferation, invasion and EMT	Cervical cancer	[46]
ALKBH5**↑**	NEAT1**↑**	ALKBH5 stabilized the transcript and facilitating NEAT1-mediated paraspeckle assembly	Facilitated metastasis	Glioblastoma multiforme	[47]
ALKBH5**↑**	NEAT1**↑**	ALKBH5 affected the m^6^A level of NEAT1	Facilitated invasion and metastasis	Gastric cancer	[49]
ALKBH5**↑**	NEAT1**↑**	ALKBH5 up-regulated NEAT1 expression	Increased proliferation and migration, repressed apoptosis	Hepatocellular carcinoma	[49]
ALKBH5**↑**	NEAT1**↑**	ALKBH5 up-regulated NEAT1 expression	Accelerated proliferation, migration, but repressed apoptosis	Colon cancer	[50]
ALKBH5**↑**	NEAT1**↑**	ALKBH5 promoted NEAT1 expression	Increased proliferation, migration, invasion, but inhibited apoptosis	Infantile hemangioma	[51]
ALKBH5**↑**	KCNQ1OT1**↑**	ALKBH5 mediated KCNQ1OT1 expression via an m^6^A-YTHDF2-dependent	Accelerated proliferation, invasion and metastasis	Laryngeal squamous cell carcinoma	[52]
ALKBH5**↑**	CASC8**↑**	ALKBH5 enhanced the stability of CASC8 transcript	Accelerated proliferation and chemoresistance	Esophageal squamous cell carcinoma	[53]
ALKBH5**↑**	RMRP**↑**	ALKBH5 enhanced the expression of RMRP	Accelerated proliferation, migration and invasion, but inhibited apoptosis	Lung adenocarcinoma	[54]
ALKBH5**↑**	TP53TG1**↓**	ALKBH5 reduced the TP53TG1 stabilization and expression	Facilitated proliferation, metastasis	Gastric cancer	[55]
ALKBH5**↑**	LINC02551**↓**	ALKBH5 downregulated LINC02551 expression	Facilitated carcinoma growth and metastasis	Hepatocellular carcinoma	[57]
ALKBH5**↑**	KCNK15-AS1**↓**	ALKBH5 increased demethylation of KCNK15-AS1 to control its elevation	Promoted proliferation, migration and EMT	Pancreatic cancer	[57]
ALKBH5**↓**	DDIT4-AS1**↑**	ALKBH5 effected the stabilization of DDIT4-AS1	Accelerated tumor growth	Pancreatic cancer	[58]
ALKBH5**↑**	PVT1**↑**	ALKBH5 associated with PVT1 and suppressed PVT1 degradation	Promoted proliferation and tumor growth	Osteosarcoma	[59]
ALKBH5**↑**	PVT1**↑**	ALKBH5 effected the expression and stability of PVT1	Accelerated tumor growth and metastasis	Lung cancer	[60]

The upward arrow represents that the elevation of expression and the downward arrow indicates the reduction of expression

## ***m***^***6***^***A modification regulated by LncRNAs in various tumors***

LncRNAs play essential roles in regulation of gene transcription and mRNA translation, implicating that LncRNA may influence m^6^A modification level through controlling methyltransferases and demethylases. Recently, accumulating studies have confirmed that LncRNA does impact the level and biological function of m^6^A modification via mediating METTL3, METTL14, WTAP, FTO and ALKBH5, and these regulatory relationships involved in the occurrence and development of various tumors, which originate from respiratory system, digestive system, reproductive system and so forth [45, 61–76].

### ***LncRNAs mediate m***^***6***^***A modification through changing methyltransferases***

#### Regulatory effects of LncRNAs on METTL3

As the active center of the methyltransferase complex, METTL3 can be regulated by LncRNAs in directly or indirectly manner. In terms of direct regulation, METTL3 expression was directly interacted with LncRNA ARHGAP5-AS1 [61], LINC00470 [19, 62] and LncRNA SNHG4 [63] in different cancer cells. Zhu et al. have indicated that targeting ARHGAP5-AS1/ARHGAP5 axis may be a promising strategy to overcome chemoresistance in gastric cancer. In detail, LncRNA Rho GTPase activating protein 5 antisense RNA 1 (ARHGAP5-AS1) has been upregulated in chemoresistant gastric cancer cells and its knockdown reversed chemoresistance. M^6^A modification on LncRNA ARHGAP5 was significantly inhibited by depletion of METTL3, which was effectively recruited by LncRNA ARHGAP5-AS1 to facilitate m^6^A modification on *ARHGAP5* mRNA [61]. Yan et al. revealed that LncRNA LINC00470 oncogenic functions on gastric cancer cell proliferation, migration and invasion were closely related to METTL3-mediated m^6^A modification on *PTEN* mRNA. In detail, an RIP assay results exhibited that METTL3 could remarkably enrich LINC00470 transcripts. Further confirmation data from RNA pull-down assay demonstrated an obvious interaction between LINC00470 and METTL3. Moreover, knockdown of LINC00470 significantly reduced the binding of METTL3 in *Phosphatase and tensin homolog (PTEN)* mRNA and depletion of METTL3 expression rescued the LINC00470-induced *PTEN* mRNA degradation, indicating LINC00470 positively regulated m^6^A modification on *PTEN* mRNA via relation to METTL3 [19]. This regulatory manner was also identified in chronic myelocytic leukemia (CML) cells, in which the alteration of LINC00470 had no effect on the luciferase activity of the PTEN promoter but affected the half-life of *PTEN* mRNA. Specifically, LINC00470 is a regulator of METTL3, which positively regulate the m^6^A modification on *PTEN* mRNA, thus enhancing the PTEN expression and stability. High expression of PTEN was able to promote protein kinase B (PKB/AKT) activity while inhibit hexokinase 1 (HK1) ubiquitination, thereby stimulating tumorigenesis of chronic myelocytic leukemia (CML) cells [62]. In addition, LncRNA SNHG4 overexpression facilitated cell proliferation and migration while inhibited cell apoptosis in neonatal pneumonia patients. Mechanistically, the enrichment of LncRNA SNHG4 in the METTL3 promoter region resulted in the downregulation of METTL3, of which interference can restrain the m^6^A modification on *STAT2* mRNA, thus promoting *STAT2* mRNA translation efficiency [63].

For the indirect regulation, LncRNAs mediate METTL3 expression mainly in a miRNA-dependent manner. In detail, Wang et al. reported that NUTM2A-AS1/miR-590-5p/METTL3 axis was involved in lung adenocarcinoma progression. Mechanically, miR-590-5p was predicted and verified as the direct target of LncRNA NUTM2A-AS1 according to bioinformatics analysis and a dual luciferase reporter assay. And further exploring data confirmed that miR-590-5p could target the *METTL3* mRNA and cut down METTL3 expression in NCI-H23 and A549 cells [64]. In addtion, the functional role of LINC00240/miR-338-5p/METTL3 axis was investigated in regulating the aggressiveness of gastric cancer cells. Specifically, LINC00240 has been identified and validated to negatively regulate miR-338-5p, which could target *METTL3* mRNA at 3'UTR to downregulate its protein translation [65].

#### Regulatory effects of LncRNAs on METTL14

Up to now, the regulatory effects of LncRNAs on METTL14 is mainly observed in breast cancer and acute myeloid leukemia (AML) [66–69]. In detail, results from Sun et al. uncovered a novel LNC942-METTL14-CXCR4/CYP1B1 signaling axis in breast cancer initiation and progression. They unveil that LINC00942 (LNC942) exerts its functions as an oncogene in promoting METTL14-mediated m^6^A modification and regulates the expression and stability of LNC942 downstream target genes C-X-C motif chemokine receptor 4 (CXCR4) and cytochrome P450 1B1 gene (CYP1B1) in breast cancer initiation and progression. Mechanistically, LNC942 directly recruits METTL14 protein by harboring the specific recognize sequence (+ 176- + 265), thereby stabilizing the *CXCR4* and *CYP1B1* mRNA in an m^6^A modification dependent manner thus finally promoting cell proliferation and colony formation of breast cancer cells [66]. LncRNA urothelial carcinoma-associated 1 (UCA1) also served as a risk factor for indicating poor prognosis of breast cancer patients and silencing LncRNA UCA1 could attenuate cell proliferation and invasion. Further exploring results showed that LncRNA UCA1 augmented the METTL14 expression through altering *METTL14* promoter region methylation, which promote the *METTL14* translation, thus mediating the miR-375 level in an m^6^A-dependent manner and increasing SOX12 expression levels and curbing the progression of breast cancer [67]. Besides breast cancer, overexpression of LncRNA UCA1 also accelerated Acute myeloid leukemia (AML) development by regulating METTL14-mediated *CXCR4* and *CYP1B1* mRNA [68].

#### Regulatory effects of LncRNAs on WTAP

As an important adaptor protein in m^6^A methyltransferase complex, WTAP can be also regulated by a variety of LncRNAs in a miRNA-dependent manner, therefore involving in the occurrence and development of various tumors [70–75]. In detail, results from Zhu et al. indicated that hypoxia upregulated the expressions of LncRNA EMS and WTAP as well as reduced the level of miR-758-3p in esophageal cancer cell line ECA-109. Further exploring data verified that targeting regulatory relationships between LncRNA EMS and miR-758-3p, as well as miR-758-3p and WTAP, implicating a critical role of LncRNA-EMS/miR-758-3p/WTAP axis in regulating hypoxia-mediated cisplatin resistance in esophageal cancer [69]. High expression of LncRNA PCGEM1 accelerates cell proliferation, migration and invasion but restrains cell apoptosis via sponging miR-433-3p to upregulate WTAP thus promoting the non-small cell lung cancer progression [70]. In hepatocellular carcinoma, functional interactions between LINC00839, miR-144-3p and WTAP were validated. In terms of specific mechanisms, LINC00839 served as a sponge to negatively regulate miR-144-3p activity, which contributed to elevation the WTAP expression [71]. A novel mechanistic role of LncRNA DUXAP8/miR-448/WTAP/Fak signaling axis has also been identified in pancreatic carcinoma [72]. In addition, Ge et al. confirmed that LncRNA SNHG10 could bind to miR-141-3p, which further targeted binding with WTAP, meaning LncRNA SNHG10 upregulated WTAP through decreasing miR-141-3p expression in osteosarcoma genesis [73].

### ***LncRNAs mediate m***^***6***^***A modification through demethylases***

#### Regulatory effects of LncRNAs on FTO

As the first identified m^6^A demethylase, FTO plays important roles in regulating the LncRNA levels and functions [45–47]. Interestingly, recent studies have found that the level of FTO is conversely regulated by LncRNAs, including LncRNA JPX [74], Lnc-H2AFV-1 [75], LncRNA CASC15 [76] and LncRNA AC026691.1 [45]. In detail, LncRNA Just Proximal to the X-inactive specific transcript (JPX) has been proven to be involved in glioblastoma multiforme through the FTO/PDK1 axis. Mechanistically, FTO interaction with LncRNA JPX, which forms a complex with *phosphoinositide dependent kinase-1* (*PDK1*) mRNA, can enhance FTO-mediated *PDK1* mRNA demethylation, thus promoting stability and translation of *PDK1* mRNA [74]. Lnc-H2AFV-1 was found to be upregulated in head and neck squamous cell carcinoma tissues, in which the expression of FTO was contrary to that of lnc-H2AFV-1. Further results revealed that lnc-H2AFV-1 overexpression led to the elevated expression and maximal m^6^A methylation of intraflagellar transport (IFT) 80 by regulating the FTO to promote HNSCC progression [75]. Qin et al. reported that inhibition of FTO expression significantly restrained proliferative and anti-apoptotic effects, which were mediated by LncRNA CASC15. Further exploring data indicated that LncRNA CASC15 promoted esophageal squamous cell carcinoma carcinogenesis by decreasing *single minded 2* (*SIM2*) stability via FTO-regulated demethylation [76]. LncRNA AC026691.1 and FTO were intimately associated with the regulation of m^6^A RNA methyladenine in gastric cancer. Combined effect of LncRNA AC026691.1 and FTO might suppress gastric cancer via downregulation of m^6^A level [45].

#### Regulatory effects of LncRNAs on ALKBH5

Up to now, two studies have reported that LncRNA CASC11 and LncRNA GAS5-AS1 can mediate the m^6^A modification through affecting ALKBH5 in hepatocellular carcinoma and cervical cancer, respectively [77, 78]. In detail, LncRNA CASC11 is involved in the regulation of cell proliferation, migration as well as invasion through the upregulation of Ubiquitin-conjugating enzyme E2T (UBE2T) in an m^6^A-dependent manner in hepatocellular carcinoma. Specificly, LncRNA CASC11 stabilized *UBE2T* mRNA through reducing *UBE2T* mRNA m^6^A modification via recruiting ALKBH5. Moreover, LncRNA CASC11 also interfered the interaction between *UBE2T* mRNA and YTHDF2, thereby influencing proliferation and metastasis of hepatocellular carcinoma cells [77]. Interestingly, LncRNA GAS5-AS1, the antisense RNA of GAS5, has been detected to interact with GAS5 and enhanced its stability through decreasing ALKBH5-mediated GAS5 m^6^A modification, therefore reducing the proliferation, migration and invasion of cervical cancer cells [78].

**Fig. 2:**
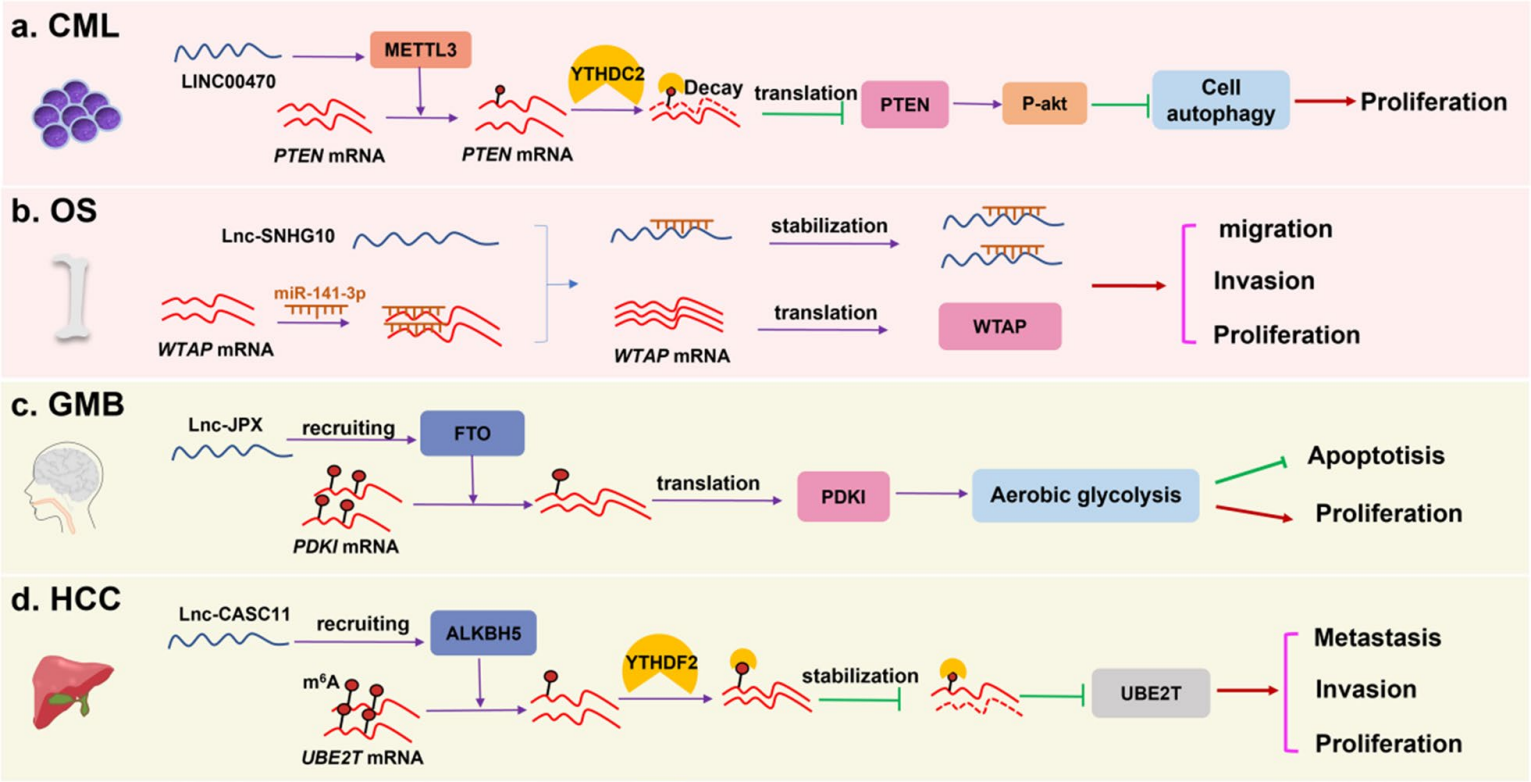
Mechanisms underlying the LncRNAs influencing m^6^A modification in multiple tumors. **a** LINC00470 reduced the PTEN expression by regulating METTL3 and promoting proliferation of chronic myeloid leukemia (CML) cells. **b** LncRNA SNHG10 sponged miR-141-3p thus upregulating WTAP expression and resulting in promoting cell proliferation in osteosarcoma genesis (OS). **c** In glioblastoma multiforme (GMB), LncRNA interacted with FTO and enhanced FTO-mediated *PDK1* mRNA demethylation, thus involving in increasing proliferation and inhibiting apoptosis. **d** In hepatocellular carcinoma (HCC), Lnc-CASC11 decreased UBE2T m^6^A modification via recruiting ALKBH5, thereby promoting proliferation, migration and invasion

Taken together, current studies have reported that LncRNAs influenced m^6^A modification through mediating catalytic enzymes, including METTL3, METTL14, WTAP, FTO and ALKBH5, during the occurrence and development of various tumors. Although specific mechanisms underlying LncRNA mediation m^6^A modification are complex, there are some similarities in LncRNA regulating m^6^A modification. We drew a schematic diagram, which takes LINC00470 [62], LncRNA SNHG10 [73], Lnc-CASC15 [76] and Lnc-CASC11 [77] as examples, to show the specific mechanisms of LncRNAs regulating the m^6^A modification through affecting m^6^A catalytic enzymes (Fig. 2). Additionally, the regulatory mechanisms of LncRNAs in m^6^A modification and its catalytic enzymes in different tumors are summarized and shown in Table 2.

**Table 2:** Regulation of m^6^A modification by LncRNAs in various tumors

LncRNAs	Regulatory proteins of m^6^A	Interactive mechanisms of m^6^A and LncRNAs	Functions	Tumors	Refs.
ARHGAP5-AS1**↑**	METTL3**↑**	LncRNA stabilized ARHGAP5 mRNA by recruiting METTL3 to stimulate m^6^A modification of *ARHGAP5* mRNA	Increased cell viability and inhibited cell apoptosis	Gastric cancer	[61]
LINC00470**↑**	METTL3**↑** YTHDF2**↑**	LncRNA -METTL3-mediated *PTEN* mRNA degradation in a YTHDF2 dependent manner	Promoted cell proliferation, migration and invasion	Gastric cancer	[19]
LINC00470**↑**	METTL3**↑**	LncRNA reduced the PTEN expression by regulated the m^6^A modification on *PTEN* mRNA through METTL3	Facilitated proliferation	Chronic myeloid leukemia	[62]
NUTM2A-AS1**↑**	METTL3**↑**	LncRNA indirect upregulated METTL3 expression	Increased proliferation, migration, invasion, metastasis	Lung adenocarcinoma	[64]
LINC00240**↑**	METTL3**↓**	LncRNA negatively regulated miR-338-5p to downregulate METTL3 expression	Accelerated invasion	Gastric cancer	[65]
LNC942**↑**	METTL14**↑**	LncRNA recruits METTL14 and stabilizes the expression of downstream targets	Promoted proliferation	Breast cancer	[66]
UCA1**↑**	METTL14**↑**	LncRNA effected the DNA methylation of METTL14 and its expression	Promoted proliferation and invasion	Breast cancer	[67]
UCA1**↑**	METTL14**↑**	LncRNA regulated METTL14-mediated *CXCR4* and *CYP1B1* mRNA	Accelerated proliferation, migration, invasion but inhibited apoptosis	Acute myeloid leukemia	[68]
EMS**↑**	WTAP**↑**	LncRNA-EMS/miR-758-3p/WTAP axis regulated hypoxia-mediated drug resistance to cisplatin	Accelerated proliferation but inhibited apoptosis	Esophageal cancer	[69]
PCGEM1**↑**	WTAP**↑**	LncRNA via sponging miR-433-3p to upregulate the level of WTAP	Accelerated proliferation, migration, invasion but inhibited apoptosis	Non-small cell lung cancer	[70]
LINC00839**↑**	WTAP**↑**	LncRNA via sponging miR-144-3p to elevated the expression of WTAP	Facilitated proliferation, migration, invasion but inhibited apoptosis	Hepatocellular carcinoma	[71]
DUXAP8**↑**	WTAP**↑**	LncRNA targeted miR-448, and miR-448 directly bound to WTAP	Promoted migration, proliferation and invasion	Pancreatic carcinoma	[72]
SNHG10**↑**	WTAP**↑**	LncRNA upregulated WTAP through decreasing miR-141-3p expression	Facilitated proliferation and migration	Osteosarcoma	[73]
JPX**↑**	FTO**↑**	LncRNA interacted with FTO and enhanced FTO-mediated *PDK1* mRNA demethylation	Facilitated proliferation and aerobic glycolysis but inhibited apoptosis	Glioblastoma multiforme	[74]
Lnc-H2AFV-1**↑**	FTO**↓**	LncRNA regulated the expression of m^6^A methylation	Strengthened proliferation	Head and neck squamous cell carcinoma	[75]
CASC15**↑**	FTO**↓**	LncRNA decreased SIM2 stability via FTO-regulated demethylation	Facilitated proliferation but inhibited apoptosis	Esophageal Squamous cell carcinoma	[76]
AC026691.1**↓**	FTO**↓**	LncRNA combined FTO thus effecting the m^6^A level	Accelerated migration and proliferation	Gastric cancer	[45]
CASC11**↑**	ALKBH5**↓**	LncRNA decreased UBE2T m^6^A modification via recruiting ALKBH5	Stimulated proliferation, migration, invasion	Hepatocellular carcinoma	[77]
GAS5-AS1**↑**	ALKBH5**↓**	LncRNA interacted with GAS5 and enhanced its stability through decreasing ALKBH5-mediated GAS5 m^6^A modification	Facilitated proliferation, migration and invasion	Cervical cancer	[78]

The upward arrow represents the elevation expression and the downward arrow indicates the reduction expression

## ***Interaction between LncRNAs and methyl-binding proteins of m***^***6***^***A modification***

As universally acknowledged m^6^A binding proteins, including YTHDFs, YTHDCs, IGF2BPs and hnRNPs, not only participate in multiple processes of LncRNA metabolism but also involve in a variety of tumorigenesis through binding to the m^6^A modification sites of target LncRNAs [79–81]. Nowadays, the interaction between LncRNAs and methyl-binding proteins of m^6^A modification has been widely considered as an important biological event during the occurrence and progression of various tumors [53, 82–111].

### Mutual regulatory effects between LncRNAs and YTH family members

YTH family members were originally identified as m^6^A methyl-binding proteins and classified into five categories, including YTHDC1, YTHDC2, YTHDF1, YTHDF2 and YTHDF3. Most of these YTH family members have been well established to interact with LncRNAs, thus involving in various cancers [82–95].

#### Interaction between YTHDC1 and LncRNAs in tumors

YTHDC1, mainly located in the nucleus, has been proven to be related to the transport, stability maintenance and degradation of various LncRNAs during tumor occurrence and development [82]. In detail, super-resolution imaging from Wang et al. revealed that the concatenated m^6^A residues on LncRNA MALAT1 acted as a scaffold for recruiting YTHDC1 to nuclear speckles. Further investigation data revealed that the recognition of m^6^A-modified MALAT1 by YTHDC1 played an essential role in maintaining the composition and genomic binding sites of nuclear speckles, which regulate the expression of several key oncogenes. More importantly, artificially tethering YTHDC1 onto m^6^A-deficient MALAT1 largely rescues the metastatic potential of cancer cells [83]. Results from Chen et al. indicated that the m^6^A-modified LncRNA TERRA formed R-loops and promoted homologous recombination, which was essential for the alternative lengthening of telomeres pathway in cancer cells. Mechanically, reduction of m^6^A modification on LncRNA TERRA by abating METTL3 or inhibition of YTHDC1 expression enhanced degradation of LncRNA TERRA, indicating the interaction between m^6^A modification and LncRNA TERRA [84]. Loss-of-function experiment demonstrated that knockdown of LINC00857 restrained cell viability, proliferation and migration as well as epithelial mesenchymal transition and strengthened cell apoptosis in colorectal cancer. Further exploring data showed that LINC00857 can be effectively bound by YTHDC1 due to the presence of m^6^A modification. Interestingly, YTHDC1 ultimately combined with *solute carrier family 7 member 5 (SLC7A5)* and increased *SLC7A5* mRNA stability to promote the proliferation and migration of colorectal cancer cells, implicating the critical role of LINC00857/YTHDC1/SLC7A5 axis in colorectal cancer progression [85]. In clear cell renal cell carcinoma cells, m^6^A-modified Lnc-LSG1, which was identified as a target of METTL14 via high-throughput methylated RNA immunoprecipitation sequencing (MeRIP-seq), can directly bind to epithelial splicing regulatory protein 2 (ESRP2) protein and then facilitate ESRP2 ubiquitination, finally resulting in ESRP2 degradation by interaction with YTHDC1 [37].

#### Interaction between YTHDF1 and LncRNAs in tumors

YTHDF1, as another YTH family member, has been evidenced to preferentially recognize m^6^A modification on LncRNAs, thus affecting the level and biological function of LncRNAs [86]. Recently research revealed that YTHDF1 involved in stability and degradation of multiple LncRNAs, such as LncRNA HCP5 [87] and LncRNA DLGAP1-AS2 [88], thus participating in regulating the progression of esophageal squamous cell carcinoma and non-small cell lung cancer, respectively. In detail, YTHDF1-regulated LncRNA HCP5 has been evidenced to involve in progression of esophageal squamous cell carcinoma. Regarding the mechanisms, LncRNA HCP5 was able to directly interact with YTHDF1, thereby strengthening the binding of YTHDF1 to *HK2* mRNA in an m^6^A- dependent manner, leading to enhancing the stability and expression of *HK2* mRNA, of which high expression is mainly responsible for carcinogenicity of esophageal squamous cell carcinoma [87]. Moreover, the interaction between LncRNA DLGAP1 antisense RNA 2 (DLGAP1-AS2) and YTHDF1 has been established to involve in non-small cell lung cancer. Further exploring data indicated that m^6^A sites on LncRNA DLGAP1-AS2 were added by METTL3 and LncRNA DLGAP1-AS2 interacted with YTHDF1 to enhance the stability of *c-Myc* mRNA through DLGAP1-AS2/YTHDF1/m^6^A/c-Myc axis [88].

#### Interaction between YTHDF2 and LncRNAs in tumors

Up to now, LncRNA FENDRR [89], LncRNA FGF14-AS2 [90], LncRNA THOR [91], LncRNA-CBSLR [92], and LncRNA STEAP3-AS1 [93], have been evidenced to interplay with YTHDF2, thus regulating the proliferation, metastasis, invasion and ferroptosis of various cancer cells. Specifically, the expression level of LncRNA FENDRR was decreased in cancerous tissues of endometrioid endometrial carcinoma patients, while the m^6^A methylation levels on LncRNA FENDRR were elevated. Further detection results demonstrated that m^6^A-modified LncRNA FENDRR was recognized by YTHDF2, which mediated LncRNA FENDRR degradation, thus promoting cell proliferation by elevating SOX4 expression in endometrioid endometrial carcinoma [89]. LncRNA FGF14-AS2 is an essential inhibitor in breast cancer metastasis and patients with high YTHDF2 and low FGF14-AS2 expression levels showed worse distant metastasis-free survival. The exploring data showed that LncRNA FGF14-AS2 is repressed by YTHDF2-regulated RNA degradation in an m^6^A-dependent manner [90]. Interestingly, LncRNA THOR can also be simultaneously combined by YTHDF1 and YTHDF2. In detail, m^6^A sites are highly enriched on LncRNA THOR transcripts and specific m^6^A readers YTHDF1 and YTHDF2 can read the m^6^A motifs and regulate the stability of the LncRNA THOR, thus mediating the proliferation, migration and invasion of cancer cells [91]. It is widely accepted that the hostile hypoxic microenvironment takes primary responsibility for the rapid expansion of tumors. A shypoxia-inducible LncRNA, LncRNA CBSLR has been identified in gastric cancer and high expression of LncRNA CBSLR protected gastric cancer cells from ferroptosis. Mechanically, LncRNA CBSLR interacted with YTHDF2 to form LncRNA CBSLR/YTHDF2/CBS signaling axis, of which activation was able to reduce the stability of *CBS* mRNA by enhancing the binding of YTHDF2 to the m^6^A-modified coding sequence (CDS) of *cystathionine beta-synthase (CBS)* mRNA [92]. Zhou et al. identified that upregulation of LncRNA STEAP3-AS1 facilitated the proliferation and metastasis of colorectal cancer cells both in vitro and in vivo. Mechanistically, LncRNA STEAP3 antisense RNA 1 (STEAP3-AS1) interacted competitively with the YTHDF2, resulting in the disassociation of YTHDF2 with *Six-transmembrane epithelial antigen of the prostate 3 (STEAP3)* mRNA, which escaped the fate of degradation mediated by m^6^A modification and elevated the protein expression of STEAP3, thus finally activation of Wnt/β-catenin signaling to promote colorectal cancer progression [93].

#### Interaction between YTHDF3 and LncRNAs in tumors

YTHDF3, another m^6^A binding protein, also interacts with different LncRNAs in NSCLC, colorectal cancer and prostatic cancer. In detail, YTHDF3 has maintained the stability of LncRNA MALAT1 in an m^6^A-dependent manner in NSCLC cells. Further detecting results indicated that m^6^A modification on LncRNA MALAT1 has facilitated formation by METTL3 [94]. YTHDF3 also has been indicated to bind to m^6^A-modified LncRNA GAS5 and facilitate LncRNA GAS5 degradation in a methylation-dependent manner, indicating a new insight into LncRNA GAS5 in colorectal cancer progression [14]. Glycolysis is a pivotal process in metabolic reprogramming of tumorigenesis. Bio-information analysis indicated that LncRNA DICER1-AS1 was downregulated in prostatic cancer and negatively correlated with glycolytic gene expression. Mechanistically, enhanced interaction between m^6^A reader YTHDF3 and LncRNA DICER1-AS1 led to degradation of DICER1-AS1 in response to glucose depletion [95].

### Mutual regulatory effects between LncRNAs and IGF2BPs in tumors

IGF2BPs, generally including IGF2BP1, IGF2BP2 and IGF2BP3, have also been identified as m^6^A methyl-binding proteins and they are involved in the stability and degradation of various LncRNAs during the tumor occurrence and development. As a binding protein of the same type, IGF2BP1, IGF2BP2 and IGF2BP3 interact with same LncRNAs in the same tumor and has the same function. For example, regulation of LncRNA FGF13-AS1 accelerated cell proliferation, migration and invasion by impairing glycolysis and stemness properties in breast cancer. Specifically, LncRNA FGF13-AS1 not only reduced the half-life of *c-Myc* mRNA by binding to IGF2BP1/2/3 but also disrupted the interaction between IGF2BP1/2/3 and *Myc* mRNA, of which high efficiency of protein translation conversely repressed LncRNA FGF13-AS1, thus forming a feedback loop of FGF13-AS1/IGF2BPs/Myc /FGF13-AS1 [96]. In addition, LncRNA MTAR1 enhanced binding between IGF2BP1/2/3 and PABP1, thereby promoting *Myc* mRNA stability [97]. Interestingly, IGF2BP1, IGF2BP2, and IGF2BP3 can also interact with different LncRNAs in various tumors and their effects on LncRNAs has great diversities [98–107].

#### Interaction between IGF2BP1 and LncRNAs in tumors

Xia et al. discovered that the global loss of LncRNA lnc-CTHCC restrained the occurrence and development of HCC. The high expression of LncRNA CTHCC in HCC benefited from increased stability, which was enhanced by METTL3-mediated m^6^A modification in an IGF2BP1/IGF2BP3-dependent manner [98]. LncRNA AC004812.2 was a protective factor in osteosarcoma and low expression of AC004812.2 predicted worse overall survival. Overexpression of AC004812.2 increased the expression levels of YTHDF1 and IGF2BP1 inhibiting 143B cell proliferation [99]. LncRNA KB1980E6.3 could facilitate breast cancer stem cells’ self-renewal and tumorigenesis under a hypoxic microenvironment both in vitro and in vivo. Mechanistically, LncRNA KB-1980E6.3 recruited IGF2BP1 to form a LncRNA KB-1980E6.3/IGF2BP1/c-Myc signaling axis that retained the stability of *c-Myc* mRNA through increasing binding of IGF2BP1 with m^6^A-modified *c-Myc coding region instability determinant (CRD)* mRNA [100]. As a tumor suppressor in multiple cancers, LINC00261 was downregulated in pancreatic cancer tissues and cell lines and high expression of LINC00261 induced cell cycle arrest and apoptosis. Specifically, LINC00261 could sequester IGF2BP1 thus inhibiting c-myc expression [101].

#### Interaction between IGF2BP2 and LncRNAs in tumors

Previous literature has reported that IGF2BP2 involves in the occurrence and development of tumors through enhancing the stability of lncRNA DANCR [103–106]. In detail, results from Hu et al. indicated that IGF2BP2 and LncRNA DANCR work together to promote cancer stemness-like properties and pancreatic cancer pathogenesis. Mechanistically, m^6^A-modified LncRNA DANCR was essential to the interaction between IGF2BP2 and DANCR, on which m^6^A modification was specifically recognized by IGF2BP2, thus promoting the LncRNA DANCR stabilization [102]. This appearance which IGF2BP2 promoted DANCR stability in an m^6^A modification dependent manner also has been evidenced in glioblastoma cells [103]. Besides LncRNA DANCR, IGF2BP2 also combined with LncRNA-PACERR, thus promoting the stability of *Kruppel-like factor 12 (KLF12)* and *c-Myc* mRNA, finally involving in elevating the number of M2-polarized cells and facilizing cell proliferation, invasion, and migration in vitro and in vivo [104]. In colorectal cancer tissues, Wang et al. reported that LncRNA LINRIS was also upregulated and LncRNA LINRIS inhibition led to impairing proliferative capability of colorectal cancer cell line. Specifically, high expression of LncRNA LINRIS promoted the IGF2BP2 stability via blocking IGF2BP2 K139 ubiquitination, which inhibited the degradation of IGF2BP2 through the autophagy-lysosome pathway [21]. In testicular cancer, data from Ye et al. indicated that LncRNA MALAT1 and IGF2BP2 were highly expressed. In terms of mechanisms, LncRNA MALAT1 contributed to testicular cancer progression through the upregulation of IGF2BP2 by binding to miR-204 [105].

#### Interaction between IGF2BP3 and LncRNAs in tumors

A novel LncRNA DMDRMR facilitated tumor growth and metastasis in clear cell renal cell carcinoma. Mechanistically, LncRNA DMDRMR bound IGF2BP3 to stabilize target genes, such as *Cyclin-dependent kinases 4* (*CDK4)*, *Collagen type VI alpha 1 (COL6A1)*, *Tumour-Derived Laminin α5 (LAMA5)*and *Fibronectin 1* *(FN1)*, in an m^6^A-dependent manner [106]. LncRNA KCNMB2-AS1 served as a competing endogenous RNA to abundantly sponge miR-130b-5p and miR-4294, resulting in the upregulation of IGF2BP3 in cervical cancer. Moreover, LncRNA KCNMB2-AS1 and IGF2BP3 formed a positive regulatory circuit that enlarged the tumorigenic effect of KCNMB2-AS1 in cervical cancer [107].

### Mutual regulatory effects between LncRNAs and hnRNPs

In addition to regulation of translation or RNA stability, the m^6^A modification can also lead to profound changes in the mRNA or LncRNA secondary structure, thus altering their interaction with proteins or RNAs, a process known as an “m^6^A switch”. As early as 2015, heterogeneous nuclear ribonucleoprotein C (hnRNPC) was identified as an “m^6^A switch” [108]. In detail, hnRNPC does not directly bind to the m^6^A modification sites but recognizes the RNA structure changed due to the presence of m^6^A modification [109]. The latest research shows that LncRNA DDX11-AS1 interacted with hnRNPC to promote Wnt/β-catenin and AKT pathways, thus promoting glioma cell proliferation and migration [110]. Subsequently, literature from Zhu et al. indicated that LncRNA cytoskeleton regulator (CYTOR) is highly expressed in Oral squamous cell carcinoma cells, and CYTOR can promote migration, invasion and epithelial mesenchymal transition in oral cancer cells. Mechanistically, nuclear-localized CYTOR interacts with hnRNPC, resulting in stabilization of *Zinc finger E-box-binding homeobox (ZEB1)* mRNAs by inhibiting the non-degradative ubiquitination of hnRNPC [111]. Another literature reported that m^6^A methylation was involved in the upregulation of LncRNA RP11. Mechanistically, a high level of m^6^A modification is able to increase binding between LncRNA RP11 and hnRNPA2B1, thus enhancing the stability of nascent LncRNA RP11 and elevating its expression in colorectal cancer cells [15]. LncRNA cancer susceptibility candidate 8 (CASC8) was highly overexpressed in esophageal squamous cell carcinoma tissues in an ALKBH5-mediated m^6^A modification dependent manner. Mechanistically, LncRNA CASC8 interacted with hnRNPL and inhibited its polyubiquitination and proteasomal degradation, thus stabilizing hnRNPL protein levels and activating the Bcl-2/caspase-3 pathway [53].

**Fig. 3:**
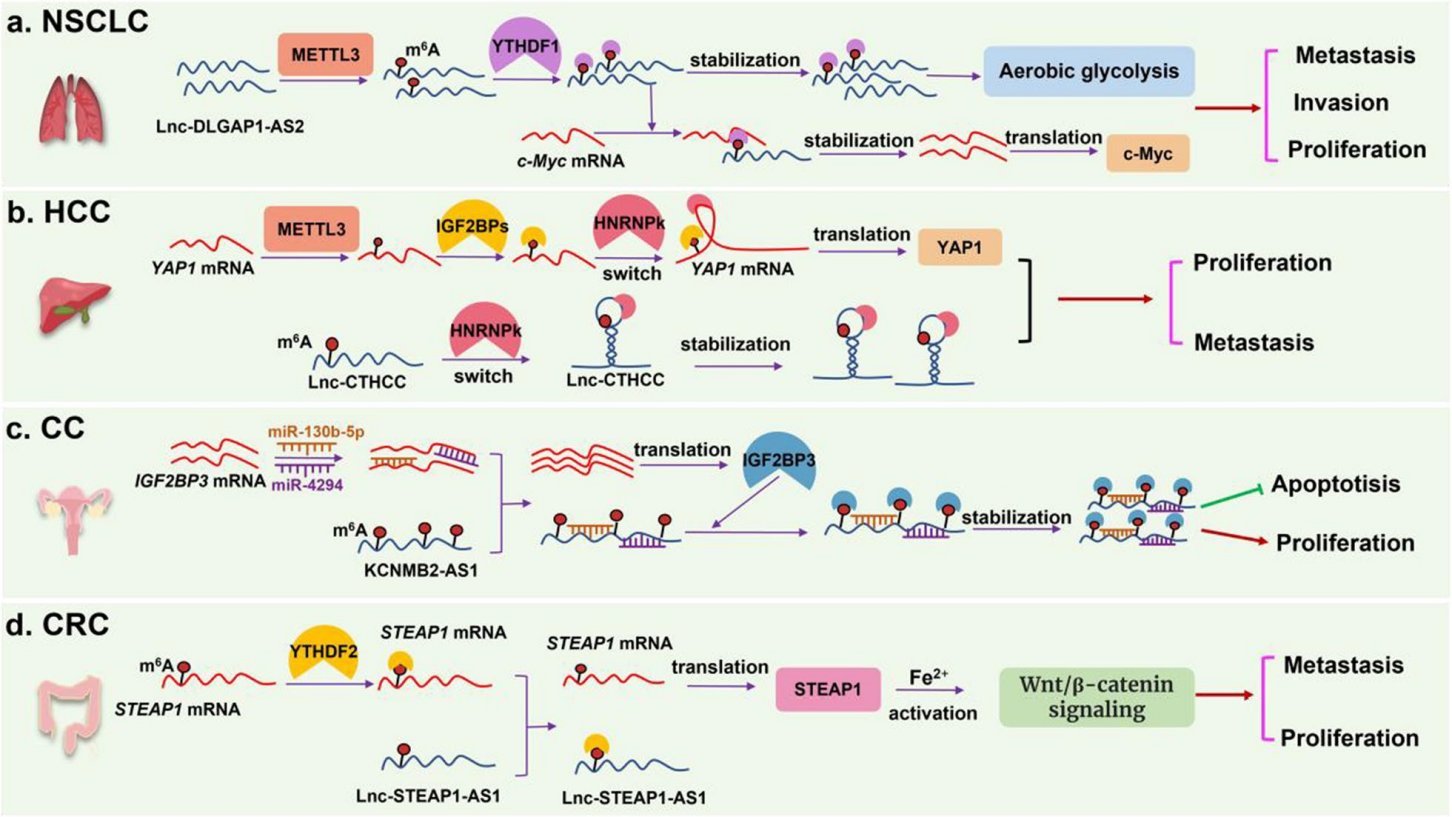
The interaction between LncRNAs and methyl-binding proteins in cancers. **a** In non-small cell lung cancer (NSCC), m^6^A modification enhanced the DLGAP1-AS2 stabilization, thus promoting Aerobic glycolysis and cell proliferation. **b** In hepatocellular carcinoma (HCC), METTL3-mediated m^6^A modification increased the stability of Lnc-CTHCC, resulting in elevating tumour growth and cell metastasis. **c** In cervical cancer (CC), Lnc-KCNMB2-AS1 up-regulation IGF2BP3 by sponging miR-130b-5p and miR-4294, resulting in increasing proliferation and restraining apoptosis. **d** In colorectal cancer (CRC), Lnc-MIR100HG competitively with YTHDF2 to elevating the protein expression of STEAP3, thus increasing cell proliferation, metastasis

In summary, emerging studies have demonstrated that the interaction between LncRNAs and methyl-binding proteins of m^6^A modification is progressively critical to the occurrence and development of various tumors. We drew a schematic diagram, which takes Lnc-DLGAP1-AS2 [88], Lnc-CTHCC [98], Lnc-KCNMB2-AS1 [107], and Lnc-STEAP3-AS1 [93] as examples, to show the specific mechanisms of the interaction between LncRNAs and methyl-binding proteins in different tumors (Fig. 3). Furthermore, the interaction mechanisms of methyl-binding proteins of m^6^A modification and LncRNAs in diverse tumors are summarized and shown in Table 3.

**Table 3:** Interaction between LncRNAs and methyl-binding proteins of m^6^A modification

M^6^A	LncRNAs	Interactive mechanisms of m^6^A and LncRNAs	Functions	Tumors	Refs.
YTHDC1**↑**	MALAT1**↑**	YTHDC1 recognized MALAT1-m^6^A and regulated oncogenes expression	Promoted metastasis	Osteoarthritis	[83]
METTL3**↓** YTHDC1**↓**	TERRA**↓**	reduction of m^6^A modification on TERRA thus enhancing degradation of LncRNA TERRA	Increased proliferation, viability	Hepatocellular carcinoma	[84]
YTHDC1**↑**	LINC00857**↑**	LINC00857 bound to YTHDC1, then YTHDC1 ultimately combined with SLC7A5 and increased its mRNA stability	Promoted cell viability, proliferation, migration, EMT, inhibited apoptosis	Colorectal cancer	[85]
YTHDC1**↑**	Lnc-LSG1**↑**	m^6^A-modified Lnc-LSG1 interaction with YTHDC1 and resulting in ESRP2 degradation	Facilitated metastasis	Clear cell renal cell carcinoma	[37]
YTHDF1**↑**	HCP5**↑**	LncRNA HCP5 interacted with YTHDF1, thereby enhancing the stability and expression of HK2 mRNA	Accelerated proliferation and invasion	Esophageal squamous cell carcinoma	[87]
METTL3**↑** YTHDF1**↑**	DLGAP1-AS2**↑**	m^6^A sites on DLGAP1-AS2 was added by METTL3 and the latter interacted with YTHDF1 to enhance the stability of c-Myc mRNA	Promoted proliferation, migration and invasion	Non-small cell lung cancer	[88]
YTHDF2**↑**	FENDRR**↓**	m^6^A-modified FENDRR was recognized by YTHDF2, which mediated LncRNA FENDRR degradation	strengthened proliferation	Endometrial carcinoma	[89]
YTHDF2**↑**	FGF14-AS2**↓**	FGF14-AS2 is repressed by YTHDF2-regulated RNA degradation in an m^6^A-dependent manner	Accelerated metastasis	Breast cancer	[90]
YTHDF1 **↑** YTHDF2**↑**	THOR**↑**	YTHDF1 and YTHDF2 read the m^6^A motifs and regulated the stability of the THOR	Enhanced proliferation, migration and invasion	Testis cancer	[91]
YTHDF2**↑** YTHDF1**↑**	CBSLR**↑**	CBSLR interacted with YTHDF2 thereby reducing the stability of CBS mRNA	Inhibited ferroptosis	Gastric cancer	[92]
YTHDF2**↓**	STEAP3-AS1**↑**	STEAP3-AS1 interacted competitively with the YTHDF2, resulting in elevating the protein expression of STEAP3	proliferation and metastasis	Colorectal cancer	[93]
YTHDF3**↑**	GAS5**↓**	YTHDF3 bound to m^6^A-modified GAS5 and facilitate GAS5 degradation in a methylation-dependent manner	Promoted proliferation and invasion	Colorectal cancer	[14]
YTHDF3**↑**	DICER1-AS1**↓**	interaction between m^6^A reader YTHDF3 and LncRNA DICER1-AS1 led to degradation of DICER1-AS1	Stimulated glycolysis, proliferation, and metastasis	Pancreatic cancer	[95]
IGF2BPs**↓**	FGF13-AS1**↓**	FGF13-AS1 not only reduced the half-life of c-Myc mRNA by binding to IGF2BPs, but also disrupted the interaction between IGF2BPs and Myc mRNA	Accelerated proliferation, migration and invasion	Breast cancer	[96]
IGF2BPs**↑**	MTAR1**↑**	MTAR1 enhanced binding between IGF2BPs and PABP1, thereby promoting Myc mRNA stability	Stimulated proliferation, migration and invasion	–	[97]
METTL3**↑** IGF2BP1**↑** IGF2BP3**↑**	CTHCC**↑**	METTL3-mediated m^6^A modification increased the stability of lnc-CTHCC	Accelerated tumour growth and metastasis	Hepatocellular carcinoma	[98]
IGF2BP1**↓** YTHDF1**↓**	AC004812.2**↓**	AC004812.2 affected the expression levels of YTHDF1 and IGF2BP1	Promoted proliferation	Osteosarcoma	[99]
IGF2BP1**↑**	KB1980E6.3**↑**	KB1980E6.3 recruited IGF2BP1 to form a axis thus retaining the stability of c-Myc mRNA	Increased self-renewal and tumorigenesis, metastasis	Breast cancer	[100]
IGF2BP1**↓**	LINC00261**↓**	LINC00261 regulated c-myc expression by sequestering IGF2BP1	Inhibited apoptosis	Pancreatic cancer	[101]
IGF2BP2**↑**	DANCR**↑**	m^6^A modification on DANCR was recognized by IGF2BP2, thus promoting DANCR stabilization	promoted proliferation and stemness-like properties	Pancreatic cancer	[102]
IGF2BP2**↑**	DANCR**↑**	IGF2BP2 promoted DANCR stability in m^6^A modification dependent manner	Accelerated the viability but decreased apoptosis	Glioblastoma	[103]
IGF2BP2**↑**	PACERR**↑**	IGF2BP2 combined with LncRNA-PACERR, thus promoting the stability of KLF12 and c-myc mRNA	Stimulated proliferation, invasion and migration	Pancreatic ductal adenocarcinoma	[104]
IGF2BP2**↑**	LINRIS**↑**	LINRIS promoted IGF2BP2 stability and inhibited the degradation via blocking IGF2BP2 K139 ubiquitination	Promoted proliferation	Colorectal cancer	[21]
IGF2BP2**↑**	MALAT1**↑**	MALAT1 upregulated IGF2BP2 by binding to miR-204	Stimulated proliferation, migration, invasion, weakened apoptosis	Testis cancer	[106]
IGF2BP3**↑**	DMDRMR**↑**	DMDRMR bound IGF2BP3 to stabilize target genes	Accelerated tumor growth, metastasis	Clear cell renal cell carcinoma	[106]
IGF2BP3**↑**	KCNMB2-AS1**↑**	KCNMB2-AS1 sponged miR-130b-5p and miR-4294, resulting the upregulation of IGF2BP3	Promoted proliferation but restrains apoptosis	Cervical cancer	[107]
HNRNPC**↑**	DDX11-AS1**↑**	DDX11-AS1 interacted with hnRNPC to promote Wnt/β-catenin and AKT pathways	Stimulated Proliferation and migration	Glioma	[110]
HNRNPC**↑**	CYTOR**↑**	CYTOR interacted with HNRNPC, resulting in stabilization of ZEB1 mRNAs	Accelerated migration, invasion, EMT	Oral squamous cell carcinoma	[111]
hnRNPA2B1**↑**	LncRNA RP11**↑**	m^6^A modification increased binding between RP11 and hnRNPA2B1, thus enhancing the stability of LncRNA and elevating its expression	Promoted migration, invasion and EMT	Colorectal cancer	[18]
HNRNPL**↑**	CASC8**↑**	CASC8 interacted with hnRNPL, thus stabilizing hnRNPL levels and activating the Bcl2/caspase3 pathway	Stimulated proliferation and chemoresistance	Esophageal squamous cell carcinoma	[53]

The upward arrow represents that the elevation of expression and the downward arrow indicates the reduction of expression

## ***The feedback loop regulation between m***^***6***^***A modification and LncRNAs in tumors***

In most studies, the attentions are on the roles of m^6^A modification regulating LncRNAs or LncRNAs mediating m^6^A modification during tumor progression, as summarized in the three sections above. Interestingly, few studies revealed that the feedback loop of m^6^A modification and LncRNAs was identified in specific cancers [112–114]. In detail, highly expression of LncRNA DLGAP1-AS1 was closely correlated to poorer clinical prognosis of breast cancer patients. Further experiment results showed that LncRNA DLGAP1-AS1 promotes ADR-resistance of breast cancer cells through a WTAP/DLGAP1-AS1/miR-299-3p/WTAP feedback loop. Mechanistically, m^6^A methyltransferase WTAP bound to the m^6^A modified sites on the LncRNA DLGAP1-AS1 and enhanced the stability of DLGAP1-AS1, which further sponged miR-299-3p and thus relieving the repression of *WTAP* mRNA [112]. Also in the breast cancer, a KIAA1429/m^6^A/LINC00667/miR-556-5p/KIAA1429 feedback loop was identified and involved in promoting the development of proliferation and migration of breast cancer cells. In detail, KIAA1429, one of the components of m^6^A methyltransferase complex, catalyzed the formation of m^6^A modification on LINC00667 and enhanced its stability. Further data revealed that highly expression of LINC00667 positively regulated KIAA1429 via sponging miR-556-5p, forming a KIAA1429/m^6^A/LINC00667/miR-556-5p/KIAA1429 feedback loop [113]. In addition, LncRNA MIR100HG has been identified as a positive regulator of EMT in colorectal cancer cells, in which hnRNPA2B1, a recognized m^6^A binding protein, was further identified as a binding partner of MIR100HG. Mechanistically, MIR100HG maintained mRNA stability of Transcription factor 7 like 2 (TCF7L2), by interacting with hnRNPA2B, which recognized the m^6^A site of *TCF7L2* mRNA in the presence of MIR100HG that may contribute to cetuximab resistance [114]_._

**Fig. 4:**
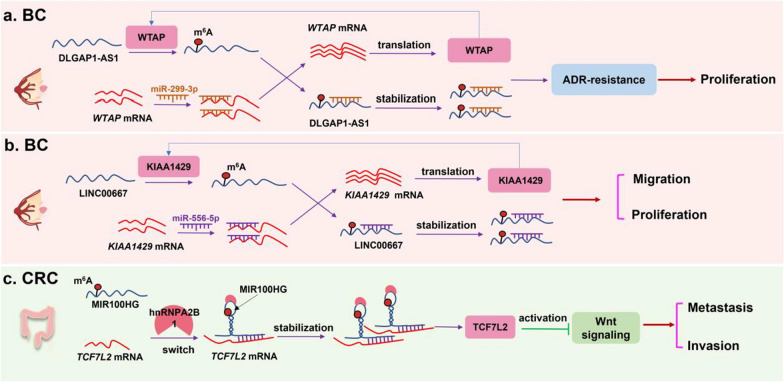
The feedback loop regulation between m^6^A modification and LncRNAs in tumors. **a** In breast cancer (BC), LncRNA DLGAP1-AS1 promotes ADR-resistance of breast cancer cells through a WTAP/DLGAP1-AS1/miR-299-3p/WTAP feedback loop, resulting in promoting cell proliferation. **b** LINC00667 positively regulated KIAA1429 via sponging miR-556-5p, forming a KIAA1429/m^6^A/LINC00667/miR-556-5p/KIAA1429 feedback loop in breast cancer, thus increasing cell proliferation and metastasis. **c** In colorectal cancer (CRC), Lnc-MIR100HG bounded hnRNPA2B1 to maintaining the *TCF7L2* mRNA stability via a MIR100HG/hnRNPA2B1/TCF7L2 regulatory axis, thereby increasing cell invasion, metastasis

We also drew a schematic diagram to show the specific mechanisms of the the feedback loop regulation between m^6^A modification and LncRNAs in breast cancer and colorectal cancer (Fig. 4).

**Fig. 5:**
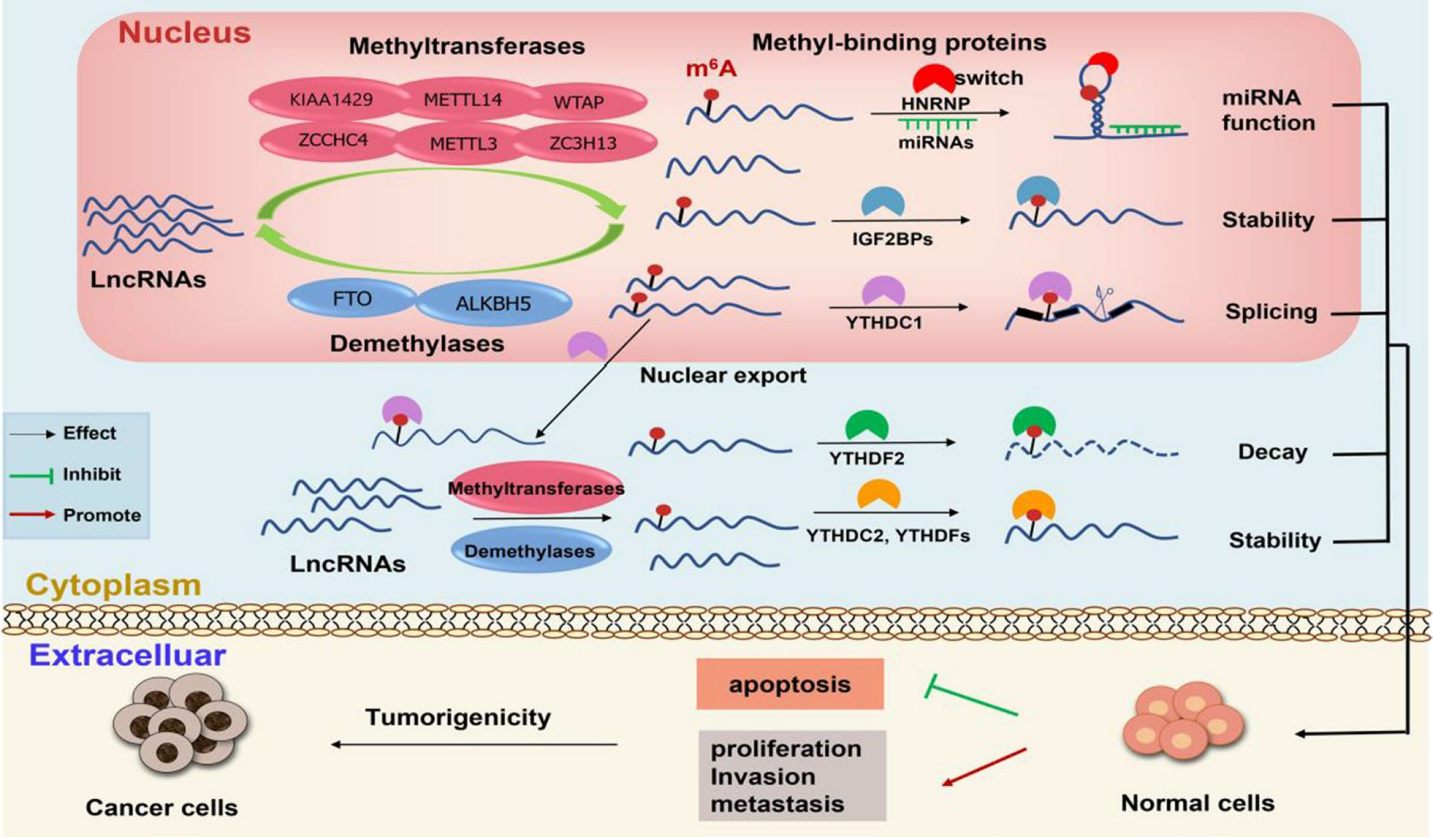
The mutual regulation between m^6^A modification and LncRNAs during tumor occurrence and development

**Fig. 6:**
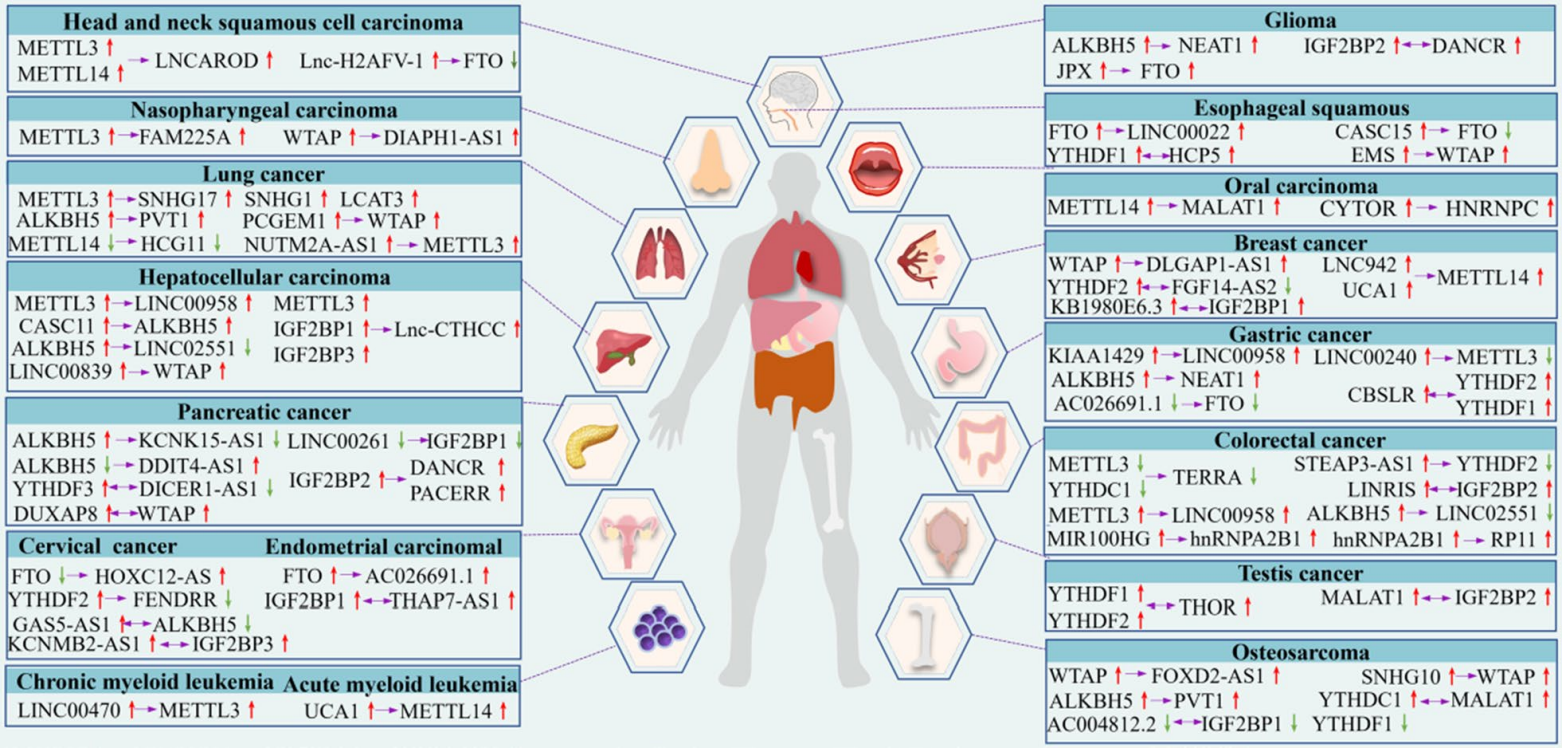
Mutual regulation between m^6^A modifications and LncRNAs. The red and green arrows respectively indicate the level rise and fall. The purple arrows point to the regulated object

In summary, m^6^A modification drives cancer cell progression, metastasis and invasion by affecting LncRNAs cleavage, stability and degradation. Meanwhile, LncRNAs have critical roles in regulating m^6^A modification through mediating its regulators during the occurrence and development of various tumors. A set of these regulatory proteins have been summarized in Fig. 5. Additionally, based on the interaction between m^6^A modification and LncRNAs, we summarized the change trends and regulation relationships between m^6^A regulatory proteins and LncRNAs in different cancers, as shown in Fig. 6.

## Conclusion and prospect

In this review, we comprehensively summarize the mutual regulation mechanisms between m^6^A modification and LncRNAs in cancer progression, metastasis, invasion and drug resistance. Firstly, m^6^A modification on LncRNAs has been shown to control corresponding LncRNA levels and functions. In detail, m^6^A modification not only affects the cleavage, stability and degradation of corresponding LncRNAs but also influences the interactions between LncRNAs and miRNAs, mRNAs or proteins, thereby regulating a variety of tumorigenesis events, such as proliferation, metastasis, invasion and apoptosis. Interestingly, LncRNAs have the potential to manipulate m^6^A modification level and biological effects through changing methyltransferases, demethylases and methyl-binding proteins of m^6^A modification, thereby affecting the occurrence and development of tumors. To be specific, LncRNA does impact the level and biological function of m^6^A modification via mediating METTL3, METTL14, WTAP, FTO and ALKBH5, and these regulatory relationships are involved in the occurrence and development of various tumors. The interactions between m^6^A modification and LncRNAs provide a new direction for exploring the potential regulatory mechanisms during carcinogenesis and contributes to targeting therapy of various tumors. Nevertheless, the present understanding of the crosstalk mechanisms between m^6^A modifications and LncRNAs may be only the tip of iceberg due to the diversity of LncRNA types and regulatory proteins of m^6^A modification. Given the critical roles of m^6^A modification and LncRNAs in tumors, further study of m^6^A and LncRNAs and their mutual regulation relationship in cancers will be worth further exploring.

## Data Availability

Not applicable.
